# Development and Acceptability of a Method to Investigate Prescription Drug Misuse in Daily Life: Ecological Momentary Assessment Study

**DOI:** 10.2196/21676

**Published:** 2020-10-01

**Authors:** Lauren M Papp, Alexandra Barringer, Shari M Blumenstock, Pamela Gu, Madison Blaydes, Jaime Lam, Chrystyna D Kouros

**Affiliations:** 1 Department of Human Development and Family Studies University of Wisconsin-Madison Madison, WI United States; 2 Department of Psychology Southern Methodist University Dallas, TX United States

**Keywords:** compliance, ecological momentary assessment, prescription drug misuse, young adult

## Abstract

**Background:**

Prescription drug misuse and abuse is an established public health challenge, and young adults are particularly affected. There is a striking lack of real-time, naturalistic data collection assessing intentions to misuse and other precipitating factors at the time of actual misuse, leaving the conditions under which individuals are most likely to misuse prescription medications unknown. Ecological momentary assessment (EMA) apps and protocols designed to capture this information would accelerate and expand the knowledge base and could directly contribute to prevention and treatment efforts.

**Objective:**

The objectives of this study are to describe the development and administration of a mobile app and the EMA protocol designed to collect real-time factors associated with college students’ prescription drug misuse intentions and behaviors in daily life; present completion rates, compliance, acceptability, and reactivity associated with the EMA protocol for participants who endorsed recent prescription drug misuse at screening (ie, risk group; n=300) and those who did not (ie, nonrisk group; n=55); and establish initial construct validity by linking the reports of misuse behaviors in daily life collected via the EMA app to prescription drug misuse reported on a standard survey.

**Methods:**

An EMA data collection app and protocol were designed specifically to capture hypothesized contextual factors along with prescription drug misuse intentions and behaviors in daily life. Using this protocol, young adult college students (N=352) completed signal- and event-contingent reports over a 28-day period. When the intention to misuse a prescription drug was endorsed, a brief follow-up prompt was sent 15 min later to collect participants’ indications of whether or not misuse had occurred.

**Results:**

Risk-group participants were significantly more likely than nonrisk counterparts to endorse any prescription drug misuse intentions in daily life (*P*<.001), to complete one or more follow-up reports (*P*<.001), and to endorse any prescription drug misuse behavior in daily life on the follow-ups (*P*<.001). Overall, participants demonstrated consistent engagement with the EMA procedures and returned an average of 74.5 (SD 23.82; range 10-122) reports. Participants in the risk and nonrisk groups did not differ in the number of reports they completed (*P*=.12), the number of their reporting days (*P*=.32), or their average completion rates (*P*=.14). The results indicated some evidence of reactivity to the momentary reporting procedure. Participants reported uniformly positive experiences and remained highly engaged throughout the reporting protocol and broader study.

**Conclusions:**

The novel EMA app and protocol provide an effective way to assess real-time factors associated with prescription drug misuse intentions and behaviors in daily life. The resulting investigations offer the potential to provide highly translatable information for research and prevention efforts.

## Introduction

### Background

Prescription drug misuse is an established public health concern in the United States and beyond [1,2]. Although available evidence, based on epidemiological studies of group averages, documents harmful correlates [3], there is a striking absence of identified antecedents related to the occurrence of prescription drug misuse in daily life by individuals with elevated risk of engaging in the behavior. This gap is surprising in light of the successful application of rigorous momentary assessment protocols to other legal and illegal substances, including alcohol, tobacco, heroin, cocaine, and cannabis [4-6]. Nonetheless, on the basis of several fundamental differences between prescription drugs and other abusable substances [7], explicit investigation of prescription medication misuse is highly justified. First, prescription medications are precisely designed for other purposes and have obvious health benefits, leading to a potential false sense of safety among people considering their misuse [8]. Moreover, prescription drugs can be taken throughout the day in nearly any setting, often without raising concerns, and their outward effects on the individual may be less noticeable. In addition, medications are frequently misused simultaneously with other substances [9], and misuse of prescriptions is identified as a major risk factor for other substance abuse [10]. Therefore, a necessary next step in this area of research is to develop a sound method to investigate the factors that contribute to prescription drug misuse in populations with elevated risks of engaging in such behavior in natural environments in daily life.

### Prescription Drug Misuse Among Young Adults: Background Research and Preliminary Study

Prescription drug misuse itself is potentially harmful and poses additional costs to individuals and society for its established links with illicit drug use, alcohol abuse, mental health problems, risky sexual behaviors, and overdose-related deaths [11-14]. College students comprise a particularly high-risk group for this behavior, with prevalence rates ranging from 5% to 43% [15,16]. College students are more likely than young adult peers to misuse stimulant medications [17] and to routinely be in situations, such as final exam periods, which involve known motivators for misuse (eg, improving study habits and grades) [18-20]. Notably, the college years during young adulthood reflect a vulnerable point in the life course for experiencing substance problems [21], and health-related functioning in this period has a unique and lasting impact on the quality of adult development [22]. Identifying the situational characteristics that predict the occurrence of prescription drug misuse would offer translatable scientific knowledge for improving young adult health.

The status quo in the literature on college students’ serious medication practices has been largely cross-sectional and based on retrospective surveys of prescription drug misuse over an extended time frame. For example, the available research predominantly involves participants indicating whether they have misused any prescription drugs over the past 12 months or their lifetime [23-26]. This global approach has offered extensive evidence on who is most likely to misuse prescription drugs and the concurrent risks associated with the misuse. However, there is a striking lack of real-time misuse data collected in natural environments on precipitating factors at the time of the misuse. This type of information allows researchers to assess the conditions under which individuals are most likely to misuse prescription medication. Findings would be critical to accelerating and expanding the knowledge base and could directly contribute to prevention and treatment efforts.

Preliminary findings have demonstrated the utility of collecting young adults’ momentary prescription drug misuse reports in real-world settings. We obtained data from 49 mixed-sex dating couples reporting 3 times per day for 10 days [27]. The approach captured misuse instances that occurred since the last report was submitted, along with participant ratings of their emotions, sexual experiences, and alcohol and other drug use. Multilevel modeling of dyadic data found that females’ prescription misuse was more likely to occur concurrently with their higher-than-average negative affect and sexual regret, whereas males’ misuse was not reliably associated with these momentary experiences. Males and females with relatively greater prescription misuse across the reporting period were more likely to engage in heavy drinking in daily life, and females with greater misuse further showed lower levels of sexual enjoyment and higher risk of unprotected sex. Thus, documenting within- and between-person correlates of young adults’ prescription drug misuse in daily life supported the present efforts to develop a more temporally precise ecological momentary assessment (EMA) design to identify real-time triggers of misuse at the time of the substance behavior and to examine initial evidence for construct validity in studies using this method.

### Objectives

Building on our preliminary work, the objectives in this study are to (1) describe the development and administration of a mobile app that was designed to collect real-time factors associated with college students’ prescription drug misuse in daily life; (2) present completion rates, compliance, acceptability, and reactivity associated with the EMA method for both risk group (n=300; endorsed recent prescription drug misuse of one or more medications in the past 3 months) and nonrisk group (n=55; did not endorse recent prescription drug misuse) participants; and (3) establish initial construct validity by linking the reports of misuse behaviors in daily life collected via the EMA app to prescription drug misuse reported on a standard survey.

## Methods

### Overview

Participants attended two laboratory sessions that were scheduled an average of 35 days apart, with the EMA protocol implemented in between these laboratory sessions. Before this study, university institutional review board approval and the National Institutes of Health Certificate of Confidentiality were obtained. Participants completed questionnaires in each laboratory session, including an EMA feedback form to describe their reporting experiences.

### Participants and Procedures

Between September 2017 and September 2019, participants at a large university in the Midwestern United States were continuously enrolled into an ongoing longitudinal study on daily behaviors and health in college life. This study is drawn from the baseline phase of a broader study [28]. Participants were recruited via flyers and announcements (eg, newspaper advertisements, emails to enrolled students) that stated, “We are particularly interested in how people use prescription medications.” Prospective participants completed a web-based screening, and a telephone call was scheduled to confirm their eligibility. Inclusion criteria for all participants were (1) being enrolled as first- and second-year college students (verified against the campus Registrar database) and (2) being 18-21 years of age; eligibility for the risk subsample also required (3) endorsing recent prescription drug misuse of one or more medications.

The web-based screening obtained prospective participants’ indications that they were in a private location and agreed to complete the screening questions, which asked about potentially sensitive day-to-day behaviors. Participants were instructed to think back over the past 3 months and indicate whether they used the medications listed in any way a doctor did not intend, such as use without a prescription, increased amounts, more often, or longer than directed. The screener presented 4 prescription medication classes adapted from the 2015 National Survey on Drug Use and Health (NSDUH) [29]: pain relievers, tranquilizers, stimulants, and sedatives or barbiturates, and common examples were provided. Prospective participants responded to each medication question (0=no; 1=yes), and multiple classes could be endorsed.

Given the main objective of capturing prescription drug misuse in daily life during this study, we oversampled for participants who endorsed recent prescription drug misuse (ie, risk group). This study included 300 participants from the risk group and 55 participants who did not endorse recent prescription drug misuse in the screener (ie, the nonrisk comparison group). To minimize any differences in recruitment between groups, the nonrisk participants were enrolled simultaneously with the risk participants throughout the recruitment period. Participants completed informed consent procedures at the start of their first laboratory session. The typical EMA reporting period was scheduled for 28 days following the first laboratory session. The length of the reporting period was adjusted for some participants due to scheduling conflicts (eg, campus recess periods) or device issues. During the second laboratory session, participants returned their devices and completed additional measures. Participants received their choice of electronic or check payments; compensation included US $75 for session 1, US $84 for reporting in daily life (prorated for partial completion), US $55 for session 2, and a US $36 bonus for maintaining compliance across the planned reporting period.

### App Development and Implementation

The app was developed in collaboration with the university’s technology division and was installed on an Apple sixth-generation iPod Touch device. Before the initiation of this study, the research team met with developers several times to provide feedback on the app features and interface design. The survey was administered through an app presented across multiple screens, with question completion automatically advancing the participant to the next screen. The questions allowed for different user inputs (eg, drop-down, radio button, and checkbox). Throughout the course of this study, the developers provided technical assistance related to software updates and other questions that arose. Developers also provided training to the research team on how to program the app and device for individual participants.

Given the sensitive nature of the data, security was a primary concern. We maximized the security of the data in several ways. First, the app was installed locally on laboratory-owned devices, which were provided to all participants for the duration of their reporting periods. Second, the data collected from the app were stored directly on the devices; the resulting data files were downloaded directly from the device to a secure server when participants returned to the laboratory (ie, never transmitted via the internet). Third, all data were recorded using a study identifier that contained no personally identifying information. Fourth, devices were also placed in restricted mode, which prevented participants from accessing a completed report or using other device functions. In addition, wireless transfer of data to and from the device was prevented. Finally, responses were all numeric and stored on the device with nondescript variable names. The list of variables was kept separate and accessible only by laboratory members. In the unlikely event someone gained access to the completed surveys, there would be no indication of what the data represented.

Consistent with numerous EMA protocols on addictions [30-33], both signal- and event-contingent assessments were administered [34]. Signal-contingent reporting involved a device prompt (ie, a notification) 4 times a day, once during each of the following periods: 8:00 AM to 11:30 AM, 11:30 AM to 3:00 PM, 3:00 PM to 7:00 PM, and 7:00 PM to 11:00 PM. The signal times within each window varied daily. Participants were also trained to self-initiate a report any time they intended to take a medication listed in any way a doctor did not direct them to use it (described under *Measures*). Signal- and event-based EMA report items were identical. Reports took approximately 2 min to complete. To reduce participant burden, a signal-contingent prompt was not sent within 2 h after a report had been completed. These EMA reports included items about intentions to misuse prescription drugs. If misuse intention of one or more of the medication classes was endorsed, participants were then sent a brief follow-up report 15 min later to assess misuse behaviors that might have occurred since the completion of the associated report; previous EMA protocols [35] have captured behaviors and contextual factors within this time frame. Acknowledging that participants might need to wait for a more convenient or private time to respond, they were instructed to complete the follow-up within 15 min. The app automatically recorded the date and time of the start and completion of all reports and follow-ups.

During the first session, participants were thoroughly trained to complete the app reporting procedures. A comprehensive definition of the focal behavior was provided (ie, using a medication without a prescription of your own; using it in greater amounts, more often, or longer than you were told to take it, or using it in any other way a doctor did not direct you to use it); the term *prescription drug misuse* was not used. Participants were trained to provide both signal- and event-contingent reports and the follow-up reports. They were also instructed to contact the laboratory staff if they experienced any problems with the device. Participants completed a practice report during their laboratory session and were instructed to carry the device at all times.

### Measures

#### Sociodemographics

During the first laboratory session, participants reported on their gender, race, and ethnicity. In addition, the participants reported fraternity or sorority affiliation and medication prescriptions they had received in their lifetime.

#### EMA and Follow-Up Reports

EMA reports captured prescription drug misuse intentions. Participants were asked “Are you about to take a medication listed here in any way a doctor did not direct you to use it?” (0=no; 1=yes). Four medication classes and examples were provided (pain relievers, tranquilizers, stimulants, and sedatives or barbiturates; 0=no; 1=yes). Reports also collected contextual information about the current location of participants and who they were with as well as hypothesized predictors of prescription drug misuse, including other substance use, mood, pain, fatigue, and stressful events. To strengthen the predictive design, reports assessed current feelings and situations; substance use covered behaviors in the past 15 min. Items were selected from brief, validated scales used in previous research with college-based populations when possible. The number of items on each report could vary because questions used conditional answer choices (eg, skip logic) whenever possible. Follow-up reports presented the following question for each of the 4 medication classes: “Have you recently taken a medication listed here, in any way a doctor did not direct you to use it?” (0=no; 1=yes). Additional questions about current mood states and recent physical activities were included in the follow-up reports.

#### Participant Feedback on the EMA Protocol (Acceptability)

During their second laboratory session, participants reported on their experiences with the app and device procedures. Participants rated the extent to which “the reporting occurred during a period that reflects my typical daily life” (0=not typical; 3=very typical) and the extent to which “the iPod touch device was user-friendly” (0=not friendly; 3=very friendly)*.* Participants rated their broader study experiences with the following: “If I contacted the lab, my questions were addressed in a helpful and timely manner” (0=not helpful; 3=very helpful) and “Would you recommend this research opportunity to your friends?” (0=no; 1=yes).

#### Past-Year Misuse of Prescription Drugs

During the baseline visit, participants completed a series of questions about the use of prescription medications following the 2015 NSDUH [29]. For this study’s 4 focal prescription medications, participants first indicated whether or not they had used any of the medications in the past 12 months. Following these initial questions, participants received a follow-up question for each endorsed medication asking if the use occurred in any way not directed by a doctor. Responses (0=no; 1=yes) were scored to reflect whether the participant engaged in any prescription drug misuse in the past year as well as any misuse of each of the 4 focal medication classes.

### Analysis Plan

We employed independent samples *t* tests and chi-square analyses to compare demographic information, completion rates, and acceptability indicators of participants in the risk and nonrisk groups. Throughout this study, we report Fisher exact test when any single cell was based on <5 participants. Reactivity was tested by correlating indicators of time and length (ie, the number of days of reporting and the number of reports completed) with the outcomes of interest (ie, reports of prescription drug misuse intentions and behavior). Initial construct validity was examined by relating past-year survey reports of prescription drug misuse assessed during the study baseline to misuse behavior on the EMA. Given that signal- and event-based EMA report items were identical (and thus were indistinguishable in the resultant data files), both types of reports were included in the completion rates and construct validity results.

## Results

### Sample Characteristics

Participants in the risk and nonrisk groups did not differ with respect to the demographic characteristics of age or class standing (Table 1). The risk-group sample included proportionally more participants who self-identified as female; non-Hispanic, White (compared with other racial or ethnic backgrounds); and affiliated with a fraternity or sorority. Risk participants were also more likely to have a lifetime history of prescriptions (of any medication class) and of pain relievers, tranquilizers, and stimulant medications.

**Table 1:** Sample characteristics at baseline and tests of risk (n=300) and nonrisk (n=55) group differences.

Characteristics			Risk participants		Nonrisk participants		Statistical comparison				*P* value
							*t* test (*df*)		Chi-square (*df*)		
Age (years), mean (SD)			19.5 (0.71)		19.36 (0.68)		1.38 (353)		N/A^a^		.17
Class standing (freshman), n %			169 (56.3)		35 (64)		N/A		1.0 (1)		.31
Sex (female), n %			207 (69)		30 (55)		N/A		4.4 (1)		.04
Race and ethnicity (non-Hispanic, White), n %^b^			240 (80.3)		32 (58)		N/A		12.7 (1)		<.001
Fraternity/sorority member (affiliated), n %			107 (35.7)		6 (11)		N/A		13.1 (1)		<.001
**Lifetime prescription history (endorsed any), n %**			207 (69)		22 (40)		N/A		17.1 (1)		<.001
	Pain reliever (endorsed)	151 (50.3)		18 (33)		N/A		5.8 (1)		.02	
	Tranquilizer (endorsed)	66 (22)		5 (9)		N/A		4.8 (1)		.03	
	Stimulant (endorsed)	53 (17.7)		2 (4)		N/A		N/A		.007^c^	
	Sedative or barbiturate (endorsed)	23 (7.7)		1 (2)		N/A		N/A		.15^c^	

^a^N/A: not applicable.

^b^Response missing for 1 risk participant.

^c^Fisher exact test.

### Data Selection and Descriptive Statistics

A total of 28,701 EMA reports were completed by 352 of the 355 participants; data were not available from 3 risk-group participants (2 did not return their data collection device and data from 1 participant’s device was not retrievable due to a password error). An important feature of the EMA protocol was collecting self-initiated reports; therefore, the app did not restrict time between reporting and allowed participants to complete several surveys within a short time frame. Upon examination of the collected data, the study team deemed it reasonable for 2 reports to be completed within 5 min of each other. Any reports beyond the second that were completed within 5 min (ie, the third or greater report) were removed. This resulted in the removal of 1.1% (329/28,701) of the obtained reports that we did not plan to include in any analysis stemming from the broader project.

Some report submissions did not adhere to the EMA protocol. Given our goal of documenting the utility of the protocol, we focus only on the reports that were obtained during the scheduled reporting periods and only the follow-ups that were completed within the instructed time frame. Although the typical reporting period was scheduled for the 28 days following the first laboratory session, some participants continued reporting until they returned for the second laboratory session. We removed a total of 2147 reports that were completed outside of the participants’ designated reporting days. The resulting 91.4% (26,225/28,701) of obtained reports were associated with 439 completed follow-ups; 64.0% (281/439) of the follow-ups were completed within 15 min of being sent (following the associated report) and were retained in this analysis.

In line with our sampling strategy, risk-group participants (143/297, 48.1%) were significantly more likely than the nonrisk counterparts (4/55, 7%) to endorse any prescription drug misuse intentions in daily life across the four medication classes (Fisher exact *P*<.001). Risk participants (108/297, 36.4%) were also significantly more likely than nonrisk participants (0/55, 0%) to complete one or more follow-up reports according to the study protocol (Fisher exact *P<*.001). In addition, risk participants (91/297, 30.6%) were more likely than nonrisk participants (0/55, 0%) to endorse any prescription drug misuse behavior in daily life on the follow-ups (Fisher exact *P<*.001).

### Compliance

Overall, participants demonstrated consistent engagement with the EMA procedures and returned an average of 74.5 reports (SD 23.82; range 10-122). Table 2 shows report completion by group status. Notably, participants in the risk and nonrisk groups did not vary in the number of reports they completed or in the number of their reporting days. We also calculated the completion rate for each person, which was based on the number of their completed reports relative to the expected number of reports (assigned number of reporting days × 4 reports per day); participants in risk and nonrisk groups did not differ in their average completion rates (Table 2).

**Table 2:** Ecological momentary assessment completion by risk (n=297) and nonrisk (n=55) group status.

Variables	Risk participants	Nonrisk participants	Statistical comparison, *t* test (*df*)	*P* value
Reports, mean (SD)	73.65 (24.07)	79.11 (22.01)	–1.57 (350)	.12
Reporting days, mean (SD)	26.50 (4.05)	27.07 (3.05)	–0.99 (350)	.32
Completion rate^a^	0.69 (0.19)	0.73 (0.18)	–1.48 (350)	.14

^a^Calculated as the number of completed reports divided by the expected number of reports based on assigned reporting days.

### Acceptability

Participants in the risk versus nonrisk groups reported similar experiences with procedures and uniformly positive experiences. As shown in Table 3, there were no reliable differences across groups in terms of evaluations of whether the reporting period was typical of their daily life or whether the device was user friendly. There was no difference in the likelihood of participants from the different groups contacting the research team for assistance; furthermore, the staff contacts that occurred were evaluated as equally helpful by participants across the groups. Nearly all participants who attended the second laboratory session (352/353, 99.7%) endorsed that they would recommend the research opportunity to their friends; 1 participant from the risk group left his or her response to this question blank.

**Table 3:** Ecological momentary assessment experiences by risk (n=298) and nonrisk (n=55) group status.

Variables		Risk participants	Nonrisk participants	Statistical comparison, *t* test (*df*)	*P* value
Reflecting typical daily life^a^, mean (SD)		2.13 (0.74)	2.33 (0.70)	–1.83 (351)	.07
User-friendly device^a^, mean (SD)		2.87 (0.37)	2.84 (0.37)	0.61 (351)	.54
**Contacted the research team, n (%)**		118 (39.6)	28 (51)	2.5 (1)^b^	.12
	Helpful contact^a^, mean (SD)	2.94 (0.27)	2.86 (0.36)	1.16 (34.76)	.25

^a^Rated on scales from 0 to 3.

^b^This result is from χ^2^, not a *t* test.

### Reactivity

We examined reactivity by testing whether prescription drug misuse intentions and behavior were more or less likely to be endorsed by participants over time, in terms of their reporting length (the number of days) and reporting amount (the number of reports). Specifically, reactivity was documented with prescription drug misuse intentions decreasing as a function of the number of reporting days (*r*=–0.01; *P*=.04) and number of reports completed (*r*=–0.03; *P*<.001). In contrast, when prescription drug misuse behavior occurred in daily life, reactivity in the opposite direction was found, with misuse behavior endorsed on the follow-up increasing as a function of reporting day (*r*=0.15; *P*=.01) and report number (*r*=0.18; *P*=.003), that is, we found evidence that prescription drug misuse intentions decreased over time (or were *less likely* to be endorsed as participants completed more days of reporting and more reports), whereas actual prescription misuse behavior increased over time (or was *more likely* to be endorsed as participants completed more reporting days and more reports). It should be noted that the magnitude of reactivity effect sizes for intentions was negligible and that for behavior was small [36].

### Initial Construct Validity

There was a robust association between participants engaging in prescription drug misuse in the past year at the baseline assessment and during the daily life procedure: Reporting misuse behavior (of one or more medication classes) in daily life on the EMA follow-up was significantly more likely among participants who had indicated any past-year prescription drug misuse (90/255, 35.3%) than among those who did not (1/97, 1%; Fisher exact *P<*.001). Examination by medication class further revealed reliable consistency in participants reporting prescription misuse behavior across the methods. Specifically, the results indicated that the proportion of participants who endorsed misuse of a medication class in daily life according to the study protocol was significantly higher among those who had reported past-year misuse of that medication class than among those who did not for all 4 medication classes: pain medication (6/23, 26% vs 2/85, 2%; Fisher exact *P*=.001), tranquilizers (9/29, 31% vs 6/79, 8%; χ^2^_1_=9.8; *P*=.002), stimulants (76/95, 80% vs 1/13, 8%; Fisher exact *P*<.001), and sedatives or barbiturates (2/7, 29% vs 1/101, 1%; Fisher exact *P*=.01).

## Discussion

### Principal Findings

This study presented the development and implementation of an app installed on study-owned devices to collect participants’ momentary experiences at the time of their prescription drug misuse intentions and behaviors. This study documented participants’ EMA completion rates and provided evidence for compliance and acceptability among participants in both risk and nonrisk sampling groups. Low levels of reactivity have been reported (described later). In addition, we presented the initial construct validity of our EMA approach by linking reports of misuse behavior in daily life to prescription drug misuse reported on a standard survey. Together, the results of this study provide early support for the feasibility and value of collecting young adults’ reports of prescription drug misuse intentions and behavior in daily life.

Although EMA still relies on self-report, this method offers improvement over more traditional assessments by minimizing reliance on memory and collecting situational factors at the time of the behavior of interest. Accordingly, these designs can provide specific momentary information to identify *more particular* risk and protective factors associated with behaviors that hold important consequences for health and adjustment. This protocol is indeed highly novel for collecting hypothesized predictors and prescription drug misuse intentions *before* the follow-up reports of the behavior. The resulting responses can allow many contextually based and temporally precise investigations across levels (ie, broader calendar-based timing as well as characteristics of the individual or the moment) to identify direct and interactive situations that increase college students’ likelihood of endorsing prescription drug misuse in daily life. Findings from our investigations, therefore, can provide highly translatable information for modifiable prevention and treatment targets.

Our sample was highly committed to the overall study and was particularly engaged with reporting in daily life. From the start of recruitment, interest in the research was high. We maintained continual interest from potential participants. Positive engagement was further reflected by nearly all participants returning to the second laboratory session with their device and saying they would recommend the experience to others. We obtained average completion rates that were similar to a pooled compliance rate on the basis of many EMA protocols that examined different substances [37]. We intentionally recruited and enrolled nonrisk participants into the study at proportionally the same rate that we enrolled the risk-group participants; therefore, academic rhythm and calendar timing were similar and broader societal and campus events that occurred were the same across these two groups. Notably, this study showed that participants from risk and nonrisk groups also had similar experiences with the reporting in daily life. Therefore, when testing hypothesized predictors of the focal substance use outcomes, we will be more confident in the findings.

The results indicated some evidence of reactivity to the momentary reporting procedure, as has been found in reporting other substances using EMA approaches (eg, alcohol) [32]. It is possible that participants were more attentive to the procedure at the beginning of their reporting periods and, therefore, were more likely to endorse misuse intentions in daily life earlier on during reporting. At the same time, it could be that reporting over time raised participants’ likelihood of engaging in the behavior or that participants became less concerned about endorsing sensitive behaviors as they become more accustomed to the reporting procedures. We are not able to determine whether reactivity varied across signal-based versus event-based reports; however, collecting such information in future studies would shed light on participants’ preferences for one type of reporting over the other. It is worth reiterating that the low magnitude of the reactivity documented for both misuse intentions and behavior suggests that the outcomes did not practically change over time as a function of having to report on behavior in real time.

In testing adherence to the method here, we conservatively focused on reports that matched protocol training time frames. The reports we plan to include in future studies with this data set will be preregistered on the Open Science Framework and will depend on particular research questions. When we investigate momentary experiences as predictors of prescription misuse behaviors in daily life, we plan to restrict analysis of the focal misuse behavior outcomes to those that were reported on follow-ups completed within 15 min of being sent (consistent with protocol training). Alternatively, studies that are designed to test background factors, or other person-level characteristics, associated with risk of engaging in prescription drug misuse in daily life would not need to be bound by these temporal restrictions. Indeed, for certain research aims, having more instances of the behavior available would result in more representative findings and more powerful statistical tests. These trade-offs should be considered and justified by the purpose of this study.

We note that our EMA reports were identical regardless of whether participants were responding to a prompt or self-initiating a report. Thus, we could not determine the completion rates for signal-based versus event-based reporting. We were also unable to establish how many participants completed event-based assessments (or the associated follow-ups). Another protocol development decision was that we did not have participants indicate retrospectively whether prescription drug misuse had occurred since their last report, that is, a coverage strategy [38]. There is a possibility that participants who become aware of the backward-looking option would be less likely to report the focal behavior in real time. This decision was particularly important for our protocol because the primary goal of the broader study was to collect momentary factors that predict real-time prescription drug misuse; any motivations or characteristics reported *after* the behavior occurred would no longer represent contextual triggers of misuse. Instead, we focused on thoroughly training participants to respond to the prompts and self-initiate as described in the protocol. Studies focused on other research questions and aims should consider whether the addition of retrospective endorsement of the behavior would bolster their design. Consistent with the development of any rigorous study, careful planning of an EMA design that matches the research question is critical.

### Limitations

Our sample was representative of the college campus from which it was drawn but is not necessarily reflective of students at other types of higher education institutions or of nontraditional students. Additional work will be needed to understand the applicability of this method to individuals across different contexts and locations. A practical limitation of using a separate device for EMA data collection is that the approach may be more burdensome for participants and requires greater investment in study materials. We expect that these considerations were offset by the anonymity and protection of the participant data provided by a separate device. Although reported behavior instances might be lower if the device was not readily available to the participant, the responses obtained may be more forthright due to security and privacy assurances. Nevertheless, in light of the recent successful apps on young adults’ own smartphones to assess drinking in daily life [32,39], this study provides a foundation for the next steps in adapting the study of prescription drug misuse in daily life to different designs. Future work could examine such possibilities.

### Conclusions

Establishing real-time mood states, pain symptoms, stressors, and other substance behaviors as predictors of college students’ prescription drug misuse is critical for obtaining a more precise understanding of the behavior in daily life situations. In line with the social ecological perspective [8,40], our models will include various triggers to identify the most robust hypothesized factor. This study provided initial evidence that using an EMA design to obtain reports of prescription drug misuse intentions and behavior (along with contextual antecedents) in daily life is feasible and acceptable. Additional research will provide direct insights into educational and intervention prospects for reducing hazardous prescription drug behaviors during an important age period.

## Abbreviations

EMA: ecological momentary assessment
NSDUH: National Survey on Drug Use and Health
